# Covalent inhibition of New Delhi metallo-β-lactamases NDM-1 and NDM-5 by 3-bromopyruvate

**DOI:** 10.1093/jacamr/dlag177

**Published:** 2026-08-25

**Authors:** Jack K Bradley, Karina Calvopina Tapia, Sabrina J Moyo, Ellinor Shore, Peter Nambala, W David Hong, Christopher J Schofield, Adam P Roberts

**Affiliations:** Centre for Drugs and Diagnostics, Department of Tropical Disease Biology, Liverpool School of Tropical Medicine, Liverpool, UK; Department of Chemistry, University of Liverpool, Liverpool, UK; Department of Chemistry and the Ineos Oxford Institute for Antimicrobial Research, University of Oxford, Chemistry Research Laboratory, 12 Mansfield Road, Oxford OX1 3TA, UK; Centre for Drugs and Diagnostics, Department of Tropical Disease Biology, Liverpool School of Tropical Medicine, Liverpool, UK; Centre for Drugs and Diagnostics, Department of Tropical Disease Biology, Liverpool School of Tropical Medicine, Liverpool, UK; Department of Clinical Sciences, Liverpool School of Tropical Medicine, Liverpool, UK; Malawi-Liverpool-Wellcome Trust Research Programme (MLW), Kamuzu University of Health Sciences, Blantyre, Malawi; Department of Chemistry, University of Liverpool, Liverpool, UK; Department of Chemistry and the Ineos Oxford Institute for Antimicrobial Research, University of Oxford, Chemistry Research Laboratory, 12 Mansfield Road, Oxford OX1 3TA, UK; Centre for Drugs and Diagnostics, Department of Tropical Disease Biology, Liverpool School of Tropical Medicine, Liverpool, UK

## Abstract

**Objectives:**

Resistance to β-lactam antibiotics, including carbapenems, mediated by metallo-β-lactamases (MBLs), including the New Delhi metallo-β-lactamase (NDM) family, is increasing. No MBL inhibitors are currently approved for clinical use, with most reported MBL inhibitors acting as metal ion chelators at either the Zn(II) ion active site and/or in solution. The hexokinase inhibitor 3-bromopyruvate (3-BP) has been reported to inhibit NDM-1. This study investigates the ability of 3-BP to restore carbapenem activity against NDM-producing bacterial strains.

**Materials and methods:**

The ability of 3-BP to restore meropenem activity was assessed against carbapenem-resistant *Escherichia coli*, *Klebsiella pneumoniae* and *Acinetobacter baumannii* strains, obtained from clinical and environmental isolates from Tanzania and Malawi. The mechanism of inhibition was investigated using mass spectrometry studies with both biologically relevant sulphur-containing compounds in addition to NDM-1 and NDM-5.

**Results and conclusions:**

3-BP selectively restored the antimicrobial activity of meropenem against resistant strains containing genes encoding NDM-1 or NDM-5 but did not restore the antimicrobial activity of amoxicillin against resistant strains containing genes encoding serine β-lactamases. Mass spectrometry studies with NDM-1 and NDM-5 support a mechanism involving covalent reaction of 3-BP with an active site cysteine residue. These results will promote work on the development of covalently reacting MBL inhibitors, a strategy that has been successful for inhibition of the nucleophilic serine β-lactamases.

## Introduction

Antimicrobial resistance (AMR) is an increasing global health problem, with an estimated 4.95 million AMR associated deaths in 2019.^1,2^ Resistance to carbapenems, once reserved as a last resort treatment against multi-drug resistant bacteria is now identified as a critical priority by the World Health Organization. The β-lactams, including penicillins, cephalosporins and carbapenems, are the most widely prescribed antibiotic class. The targets of β-lactams are transpeptidases (penicillin-binding proteins, PBPs), responsible for the crosslinking of peptidoglycan during bacterial cell wall biosynthesis.^3^ Inhibition of PBPs prevents the protein from performing its normal function, blocking crosslinking and resulting in weakening of the cell wall and autolysis.^4,5^ An important mechanism of resistance to β-lactams involves their β-lactamase catalysed hydrolysis, rendering the antibiotic inactive.^6,7^ Ambler class A, C and D β-lactamases are nucleophilic serine enzymes, with the class B metallo β-lactamases (MBLs) requiring one or two active site Zn^2+^ ions that activate a water molecule for β-lactam hydrolysis. MBLs are divided into subclasses B1–B3 based on the number of metal ions found in the active site as well sequence and structural similarities, with the B1 MBL subfamily currently being the most clinically relevant.^8,9^

Following the discovery of the subclass B1 MBL New Delhi metallo-β-lactamase-1 (NDM-1), NDM-1 and its multiple variants have emerged as one of the most prominent and clinically relevant MBL subfamilies.^10,11^ They are found to confer resistance to nearly all β-lactams, including carbapenems.^12–14^ NDMs are now prevalent in Asia, the Middle East, Europe, Africa and the Americas, largely driven by mobile plasmids that transfer between Gram-negative bacteria such as *Escherichia coli*, *Klebsiella pneumoniae* and *Acinetobacter baumannii*.^15^ The active site of NDM-1 uses two Zn(II) ions for catalysis. In the NDM zinc-1 site, the zinc ion is coordinated by three histidine residues, while at the zinc-2 site, the zinc ion is coordinated to by a cysteine, a histidine and an aspartic acid residue.^16^ The cysteine residue is conserved across B1 MBL active sites and may possess sufficient nucleophilicity to enable covalent targeting.

Clinically, serine β-lactamase inhibitors (e.g. clavulanic acid) work by covalently reacting mechanisms and are used in combination with β-lactam antibiotics to overcome resistance, by contrast, no clinically useful MBL inhibitors are currently available.^17,18^ MBLs are challenging targets due to their structural diversity and the presence of structurally related human MBL fold enzymes.^10,19^

Most reported MBL inhibitors are metal ion chelators, acting either at the Zn(II) ion active site and/or in solution, although alternative approaches based on covalent modification of cysteine residues have emerged.^20–23^ Notably, ebselen provided the first proof of concept for a covalent inhibition approach through the formation of a stable Se–S bond with the cysteine found in the MBL catalytic site, an approach that that been further explored through the development of selenium containing covalent MBL inhibitors derived from cephalexin.^21,22^ While these compounds covalently bind through a selenium moiety, the hexokinase inhibitor 3-bromopyruvate (3-BP) is another exception, with its inhibitory activity linked to the presence of an electrophilic halomethyl ketone.^20,24^ 3-BP is an small electrophilic halomethyl ketone derivative of the cellular metabolite pyruvate and has previously been explored as a potential anti-cancer treatment.^25^ 3-BP has demonstrated activity in a number of animal tumour models and has been used in clinical trials.^26,27^ These positive results, combined with low cytotoxicity versus mouse fibroblastic cells at concentrations up to 200 μM, suggests 3-BP is a potentially safe lead for therapeutic development.

3-BP is also reported to inhibit NDM-1, restoring the activities of meropenem and cefazolin against *E. coli* strains producing NDM-1 (at 64 mg/L).^20^ 3-BP was shown to inhibit subclass B1 MBLs (NDM-1, VIM-2, IMP-1) and B2 (ImiS) MBLs, but not the B3 MBL L1, in accord with the presence of an active site cysteine residue in the B1 and B2 MBLs, but not the B3 MBLs.^20,28^

Here, we report that moderate concentrations of 3-BP restore the activity of meropenem at the susceptibility breakpoint (2 mg/L) against clinically and environmentally derived resistant Enterobacterales isolates containing MBLs NDM-1 and NDM-5. 3-BP, however, did not restore activity of amoxicillin at the susceptibility breakpoint (8 mg/L) against environmentally derived Enterobacterales isolates containing SBLs TEM-1B, CTX-M-15, ACT-16, OXA-1 or OXA-48. The parent scaffold pyruvic acid showed no activity against isolates containing MBLs or SBLs, with the introduction of bromine in 3-BP leading to the observed inhibition. Small molecule mass spectrometry (MS) studies, followed by denaturing mass spectrometry studies on apo- and metallated-forms of NDM-1 and NDM-5 to investigate the mechanisms of inhibition by 3-BP, identifying the formation of covalent adducts.

## Materials and methods

Mueller–Hinton broth, amoxicillin and meropenem were from Fisher Scientific. All AST methods follow European Committee on Antimicrobial Susceptibility Testing (EUCAST) guidelines. Bacterial strains were stored in glycerol stocks at −70°C. 3-BP and pyruvic acid (>98% purity) were from Sigma Aldrich. Cysteine and glutathione (>95% purity) were from Fluorochem.

### Broth microdilution assays

Selected bacterial strains were grown from glycerol stocks on Mueller–Hinton agar for 16–18 hours overnight at 37°C. Selected colonies were then suspended in 10 mL Mueller–Hinton broth and allowed to grow for 16–18 hours overnight at 37°C. The optical density (OD) of the inoculum was measured and then the inoculum diluted to give an optical density of 1 (OD1) at a 600 nm wavelength. OD1 was then further diluted 1/1000 in Mueller–Hinton broth to obtain an inoculum suspension equivalent to for a single drug minimum inhibitory concentration (MIC) experiment or 1/500 inoculum suspension equivalent for a rescue concentration (RC) experiment. Once prepared the inoculum was used within 30 minutes. Serial dilution was performed from wells 2–10 to obtain final inhibitor concentrations from 256 to 1 mg/L with well 11 acting as a control (no drug). To determine the RC of 3-BP and pyruvic acid, meropenem or amoxicillin was then added to each well at a final concentration of 2 or 8 mg/L, respectively, when combined with 3-BP or pyruvic acid. After inoculation, the 96-well plates were incubated at 37°C for 16–18 hours. All drugs were tested via three biological replicates and three technical replicates performed. MIC/RC was recorded at the lowest concentration of no visible growth of bacteria.

### Mass spectrometry assays

#### Small molecule mass spectrometry studies

For small molecule MS studies 10 mM stock concentrations of 3-BP, glutathione and cysteine were made using HPLC grade water. 2 mL of 3-BP was added to 2 mL volumes of glutathione and cysteine and allowed to stir for 18 hours at 37°C. After 18 hours, the reaction mixture was filtered and submitted for mass spectrum analysis. High resolution mass spectra were obtained using an Agilent QTOF 6500 series spectrometer, equipped with an electrospray ionization interface and operated in the positive mode. Compact mass spectra were obtained using an Advion Expression CMS with an electrospray ionization interface and operated in negative ion mode.

#### Preparation of the metal-depleted enzyme

The zinc content of the stock enzyme preparation was depleted by dialysis in a MINI dialysis device (0.5 mL, 10 kDa molecular weight cut off) against a buffer containing 50 mM HEPES (pH 7.2), 30 mM EDTA and 5 mM phenanthroline. MBL protein was subsequently dialysed against 50 mM HEPES (pH 7.2), 1% Chelex resin to remove free metal ions of metals and phenanthroline. Following dialysis, the protein was concentrated using an Amicon^®^ Ultra Centrifugal Filter (0.5 Ml, 10 kDa molecular weight cut off).

#### ESI mass spectrometry

Purified NDM-1/NDM-5 and metal-depleted NDM-1/NDM-5 stocks were diluted to 1 μM and 10 equivalents of 3-BP was added to each sample. Samples were analysed by LC–ESI MS using a Xevo G2-S mass spectrometer coupled to an Acquity UPLC system (Waters) fitted with a ProSwift RP-4H column (1 × 50 mm; Thermo Fisher Scientific). Proteins were loaded in 95% water, 5% acetonitrile and 0.1% formic acid, and eluted over a 10-min gradient to 95% acetonitrile containing 0.1% formic acid before direct introduction into the ESI source. Protein retention times were ∼4–5 min. Data were processed using MassLynx 4.1 (Waters), and spectra were deconvoluted using the MaxEnt1 algorithm.

#### Protein modelling

AlphaFold server (version 3) was used for automated protein tertiary structure modelling of apo- NDM-1 and NDM-5.^29^ Modelling results were visualized using UCSF Chimera X 1.10.1.^30^

## Results and discussion

### Minimum inhibitory concentration and rescue concentration of 3-BP against MBL and SBL producing isolates

To investigate whether 3-BP restored antimicrobial activity of β-lactam antibiotics against bacterial strains producing NDM-1 and NDM-5, MICs of meropenem, pyruvic acid and 3-BP were determined (Table 1) according to the EUCAST guidelines.^31^ Clinical and environmental isolates of *A. baumannii*, *K. pneumoniae* and *E. coli* producing NDM-1 or NDM-5 were obtained during studies investigating neonatal diseases in Tanzania.^15,32,33^ Additional isolates were obtained through the National Collection of Type Cultures and noted by their NCTC number or from an ongoing hospital wastewater surveillance study (STRESST) based at the Queen Elizabeth Central Hospital, Blantyre, Malawi. It is notable that genes coding for serine β-lactamases are also in the different tested isolates, including *bla*_ADC_, *bla*_CARB_, *bla*_CTX_, *bla*_TEM_ and *bla*_OXA_ (Table S1, available as Supplementary data at *JAC-AMR* Online).^15,32^

**Table 1.** Microbiological comparison of meropenem, pyruvic acid and 3-BP activities against NDM-1 or NDM-5 containing *A. baumannii*, *K. pneumoniae* and *E. coli* strains

MIC (mg/L)											
	*A. baumannii*			*K. pneumoniae*				*E. coli*			
Compound	NCTC12156 (No MBL)	DT0544 (NDM-1)	DT01139 (NDM-1)	NCTC13442 (No MBL)	NTC13443 (NDM-1)	ArmMLGA211 (NDM-1)	ESSCAI122 (NDM-1)	SM247682033 (No MBL)	MLGA91 (NDM-5)	MLGA92 (NDM-5)	ESCAI231 (NDM-5)
Meropenem	<1	128	128	<1	>256	128	128	<1	128	128	64
Pyruvic acid	>256	>256	>256	>256	>256	>256	>256	>256	>256	>256	>256
3-BP	>256	>256	>256	>256	>256	>256	>256	>256	>256	>256	>256

RC determined at the susceptibility breakpoint of meropenem (2 mg/L) (mg/L)											
	*A. baumannii*			*K. pneumoniae*				*E. coli*			
Compound	NCTC12156 (No MBL)	DT0544 (NDM-1)	DT01139 (NDM-1)	NCTC13442 (No MBL)	NTC13443 (NDM-1)	ArmMLGA211 (NDM-1)	ESSCAI122 (NDM-1)	SM247682033 (No MBL)	MLGA91 (NDM-5)	MLGA92 (NDM-5)	ESCAI231 (NDM-5)
Pyruvic acid	<1	>256^a^	>256	<1	>256	>256	>256	<1	>256	>256	>256
3-BP	<1	32	32	<1	128	32	64	<1	32	32	16

RC: minimum concentration required for no visible growth of bacteria when used synergistically with meropenem set at a concentration of 2 mg/L. Concentrations of pyruvic acid and 3-BP needed to restore the activity of meropenem at its EUCAST susceptibility breakpoint concentration of 2 mg/L in NDM-1 or NDM-5 containing *A. baumannii*, *K. pneumoniae* and *E. coli* strains. Plates were incubated at 37°C for 16–18 h. Three biological and three technical replicates were performed.

^a^RC value of >256 mg/L corresponds to no observable recovery of meropenem activity at 2 mg/L.

Control isolates without MBLs were found to be susceptible to treatment with meropenem, with an MIC of <1 mg/L being observed. By contrast, isolates with genes encoding for the MBLs NDM-1 or NDM-5 demonstrated a marked reduction in susceptibility to meropenem. For the meropenem resistant strains, MIC values ranged from 64 to 128 mg/L, with one isolate exhibiting an MIC > 256 mg/L. When the isolates were treated with pyruvic acid or 3-BP alone, no measurable inhibitory activity was observed (>256 mg/L), indicating a lack of intrinsic antimicrobial activity of these two compounds.

It was observed that 3-BP was able to restore the antibacterial activity of meropenem, set at the EUCAST susceptibility breakpoint of 2 mg/L against resistant isolates and reported as the RC (Table 1). In general, a RC of 32 mg/L 3-BP was required to restore the activity of meropenem against the tested isolates, independent of the presence of NDM-1 or NDM-5. It was previously reported that 3-BP showed inhibition of NDM-1 in cell-based assays, with 3-BP (64 mg/L) restoring the activity of both meropenem and cefazolin against *E. coli* strains producing NDM-1.^20^ Our observations show that 3-BP exhibits enhanced activity against NDM-1 producing *A. baumannii* isolates (DT0544 and DT01139) and NDM-5 producing *E. coli* isolates (MLGA91, MLGA92 and ESCAI231), requiring 32 mg/L 3-BP to restore meropenem activity. *Klebsiella pneumoniae* isolates required the highest concentration of 3-BP to restore meropenem activity, with two out of the three MBL containing isolates requiring >32 mg/L 3-BP, with NCTC13443 requiring 128 mg/L and ESSCAI122 requiring 64 mg/L 3-BP to restore meropenem activity. Pyruvic acid, the parent scaffold of 3-BP displayed no significant activity against any of the isolates containing genes encoding for NDM-1 or NDM-5, consistent with reported results against NDM-1.^20^ These observations support the proposal that the B1 MBL inhibitory activity of 3-BP is due to the reactivity of its electrophilic halomethyl ketone.

3-BP was also investigated as a potential SBL inhibitor, with MICs of amoxicillin, pyruvic acid and 3-BP determined according to the EUCAST guidelines (Table 2).^31^ Isolates of *Enterobacter cloacae*, *K. pneumoniae* and *E. coli* producing at least one of following SBLs; TEM-1B, CTX-M-15, ACT-16, OXA-1 or OXA-48 were tested against (Table S2).^33^ Additional isolates were obtained through the National Collection of Type Cultures and noted by their NCTC number.

**Table 2.** Microbiological comparison of amoxicillin, pyruvic acid and 3-BP activities against SBL containing *E. cloacae*, *K. pneumoniae* and *E. coli* strains

MIC (mg/L)					
	*A. baumannii*	*K. pneumoniae*	*E. coli*		
Compound	SM247691861A (ACT-16)	NCTC13442 (TEM-1B, OXA-48, SHV-89)	SM247682033 (TEM-1B)	SM247592071 (TEM-1B, CTX-M-15)	SM247601654 (CTX-M-15, OXA-1)
Amoxicillin	256	256	256	256	256
Pyruvic acid	> 256	> 256	> 256	> 256	> 256
3-BP	> 256	> 256	> 256	> 256	> 256

RC determined at the susceptibility breakpoint of amoxicillin (8 mg/L) (mg/L)					
	*A. baumannii*	*K. pneumoniae*	*E. coli*		
Compound	SM247691861A (ACT-16)	NCTC13442 (TEM-1B, OXA-48, SHV-89)	SM247682033 (TEM-1B)	SM247592071 (TEM-1B, CTX-M-15)	SM247601654 (CTX-M-15, OXA-1)
Pyruvic acid	> 256^a^	> 256	> 256	> 256	> 256
3-BP	> 256	> 256	> 256	> 256	> 256

RC: minimum concentration required for no visible growth of bacteria when used synergistically with amoxicillin set at a concentration of 8 mg/L. Concentration of pyruvic acid and 3-BP needed to restore the activity of amoxicillin at its EUCAST susceptibility breakpoint concentration of 8 mg/L in SBL containing *E. cloacae*, *K. pneumoniae* and *E. coli* strains. Plates were incubated at 37°C for 16–18 h. Three biological and three technical replicates were performed.

^a^RC value of >256 mg/L corresponds to no observable recovery of amoxicillin activity at 8 mg/L.

Isolates bearing SBLs manifested resistance to amoxicillin, with isolates showing an MIC of 256 mg/L, in contrast to the observed meropenem susceptibility (MIC of <1 mg/L) of the strains NCTC13442 and SM247682033. When the isolates were treated with pyruvic acid or 3-BP alone, no measurable activity was observed (>256 mg/L), again indicating a lack of intrinsic antimicrobial activity. Neither pyruvic acid nor 3-BP restored the activity of amoxicillin set at the EUCAST susceptibility breakpoint of 8 mg/L, with MIC values exceeding 256 mg/L (Table 2). These observations suggest that 3-BP inhibitory activity is selective towards MBLs.

This observed selectivity towards MBLs over SBLs shows promise relating to off-target reactivity, which represent a challenge in 3-BPs clinical translation. 3-BP has pronounced electrophilic character, which has been previously reported to form a stable glutathione conjugate, reducing the amount of free inhibitor available for target engagement.^34,35^ In addition to glutathione, 3-BP has been reported to inhibit other cysteine-dependent enzymes, including hexokinase II (HKII) and glyceraldehyde-3-phosphate dehydrogenase (GAPDH).^24,36,37^ However, these observations highlight 3-BPs propensity to form conjugates with sulphur-containing compounds, giving explanation as to why 3-BPs inhibitory activity is selective towards cysteine containing MBLs.

### Mass spectrometry studies

**Figure 1.**
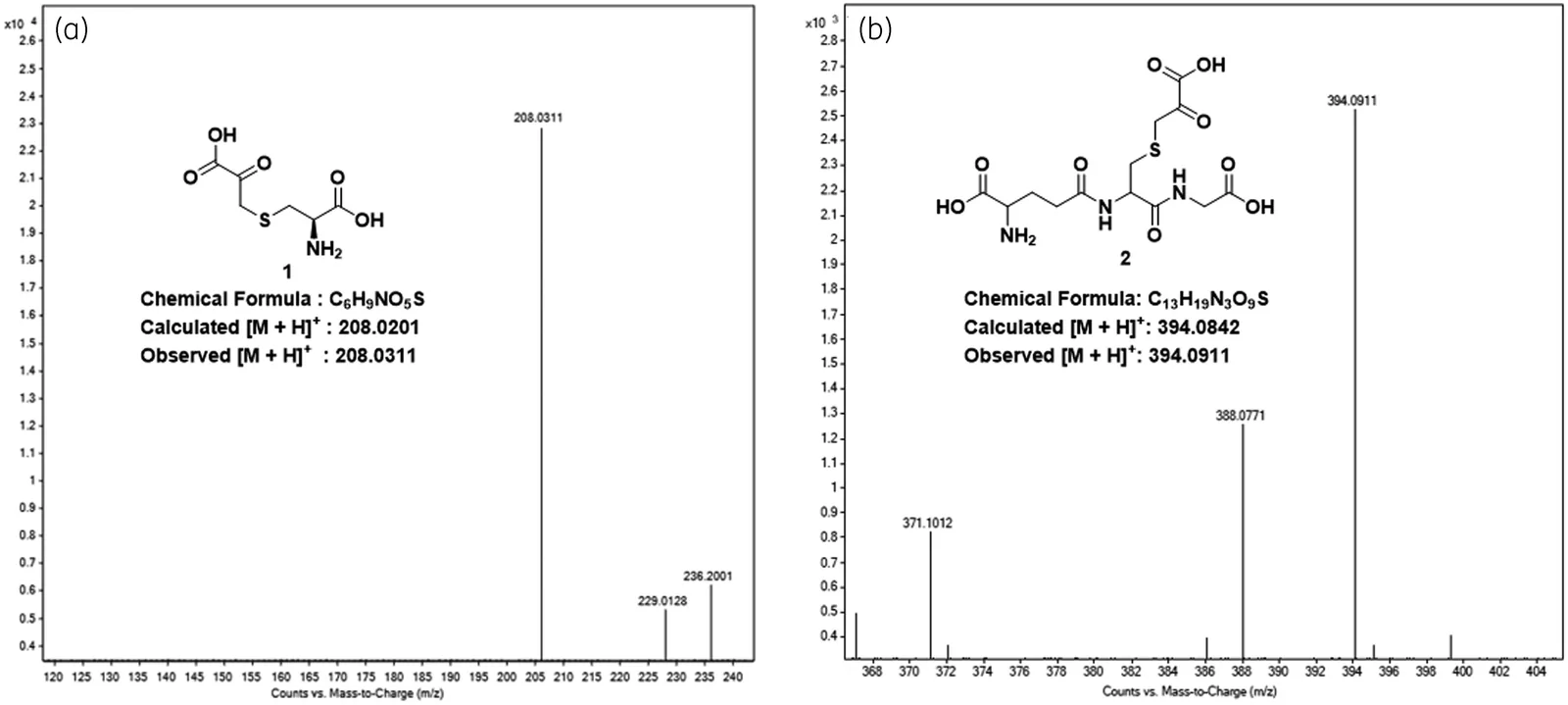
Small molecule MS assays between 3-BP (165.9266 Da/167.9245 Da), cysteine (121.0197 Da) and glutathione (307.0838 Da) were performed. Observations show reaction of 3-BP (10 mM) conjugation with cysteine (10 mM) [M + H]^+^ - 208.0311 Da (a) and glutathione (10 mM) [M + H]^+^ - 394.0911 Da (b), under conditions used to replicated those of MIC and RC assays (37°C, 16–18 h).

To investigate the role of the active site cysteine in the covalent binding of 3-BP to MBLs, initial small molecule MS studies were performed against cysteine and glutathione to ascertain whether 3-BP readily forms a covalent bond with biologically relevant sulphur-containing compounds as previously reported, under conditions used to replicated those of MIC and RC assays (37°C, 16–18 h).^34^ As anticipated, MS studies demonstrated the reaction of cysteine and glutathione with 3-BP, giving S-alkylated products. Two conjugate mass peaks of 208.0311 Da and 394.0911 Da were obtained respectively (Figure 1). The formation and subsequent stability of the thioether conjugate of 3-BP (10 mM) with glutathione (10 mM) was further investigated through additional LC-MS analysis in negative mode (Figure S1). Under conditions used to replicated those of MIC and RC assays (37°C, 16–18 h), the formation and stability of the thioether conjugate was assessed at 5 minutes, 15 minutes, 1 hour, 18  hours and 48 hours. It was observed that after 5 minutes the thioether conjugate started to form ([M - H]^−^ 392 Da), with glutathione still present as the major fragment ([M - H]^−^ 306 Da). Within 15 minutes of 3-BP addition, glutathione levels were depleted, with the major fragment observed as the thioether conjugate ([M - H]^−^ 392 Da), consistent with previous findings.^35^ This thioether conjugate remained stable up to 48 hours after initial addition under these conditions, showing good stability and reinforcing 3-BPs potential to target sulphur-containing compounds.

**Figure 2.**
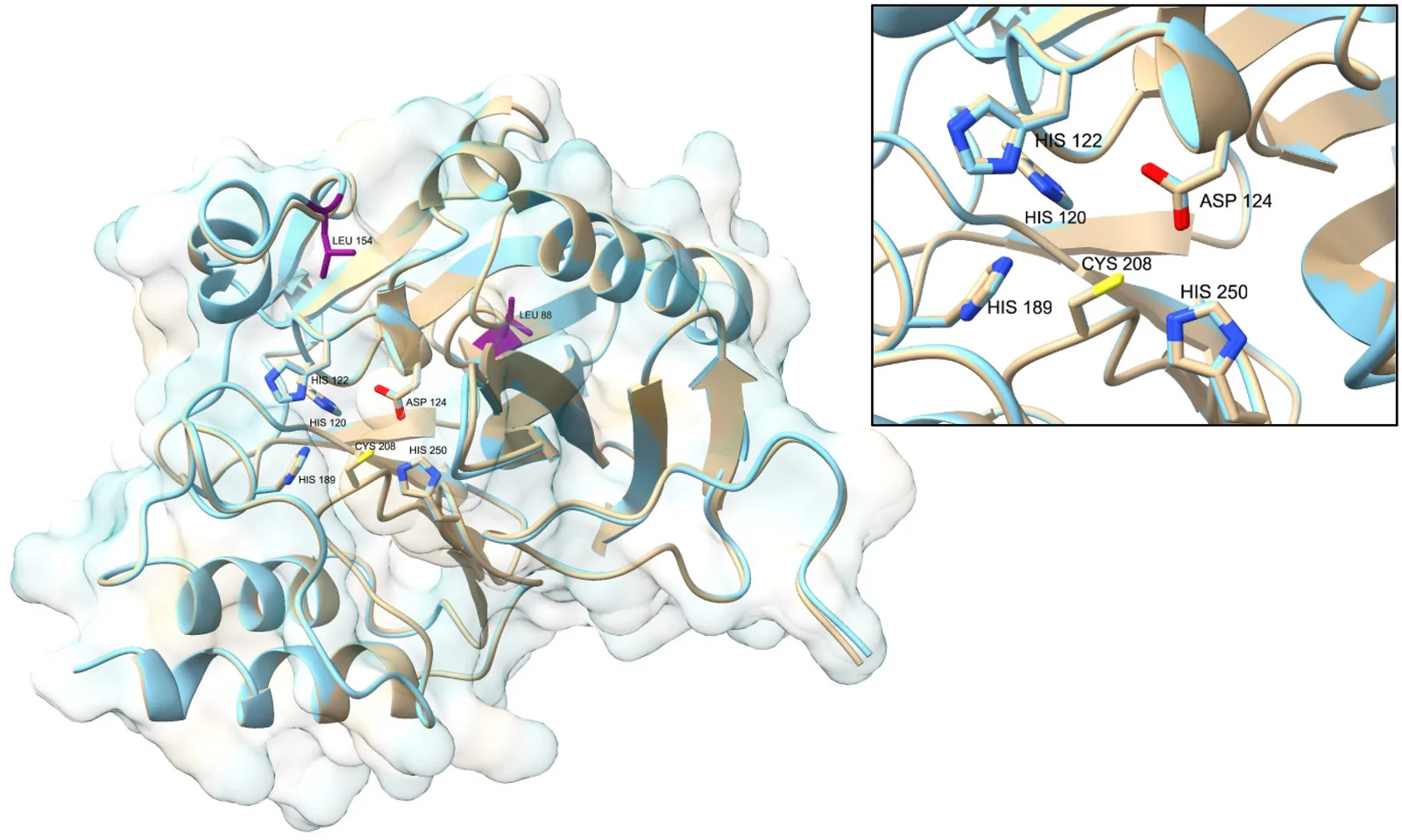
Comparison of AlphaFold modelled apo-NDM1 (brown) and NDM-5 (blue). Overlayed views show high structural similarity with changes in NDM-5 highlighted in purple, (Val88Leu and Met154Leu). Insert, expansion of the apo- NDM-1 (brown) and NDM-5 (blue) active sites. The zinc-1 site is defined by three histidine residues while the zinc-2 site is defined by a cysteine, histidine and aspartic acid residue.

The reaction of 3-BP with Zn(II) complexed- and apo- (non-zinc containing) NDM-1 and NDM-5 was investigated using denaturing electrospray ionization MS conditions. When comparing the amino acid sequences of each MBL, only one cysteine can be found in each enzyme, meaning that if any 3-BP were to bind with a solvent accessible nucleophile, it would bind at the Cys(208) position within the active site and not at an allosteric site. AlphaFold was used for tertiary protein structure modelling of the two apo- proteins.^29^ It was found that both NDM-1 and NDM-5 shared the same B1 MBL subclass active site as expected, with NDM-5 varying from NDM-1 through two amino acid substitutions at position 88 (Val-Leu) and position 154 (Met-Leu) as highlighted in purple (Figure 2). These substitutions have resulted in NDM-5 showing an increased resistance and stability under conditions where zinc is depleted.^38,39^ This was observed during buffer apo-enzyme preparation, where it was noted that NDM-1 precipitated from solution in the absence of zinc, yet NDM-5 remained in solution.

**Figure 3.**
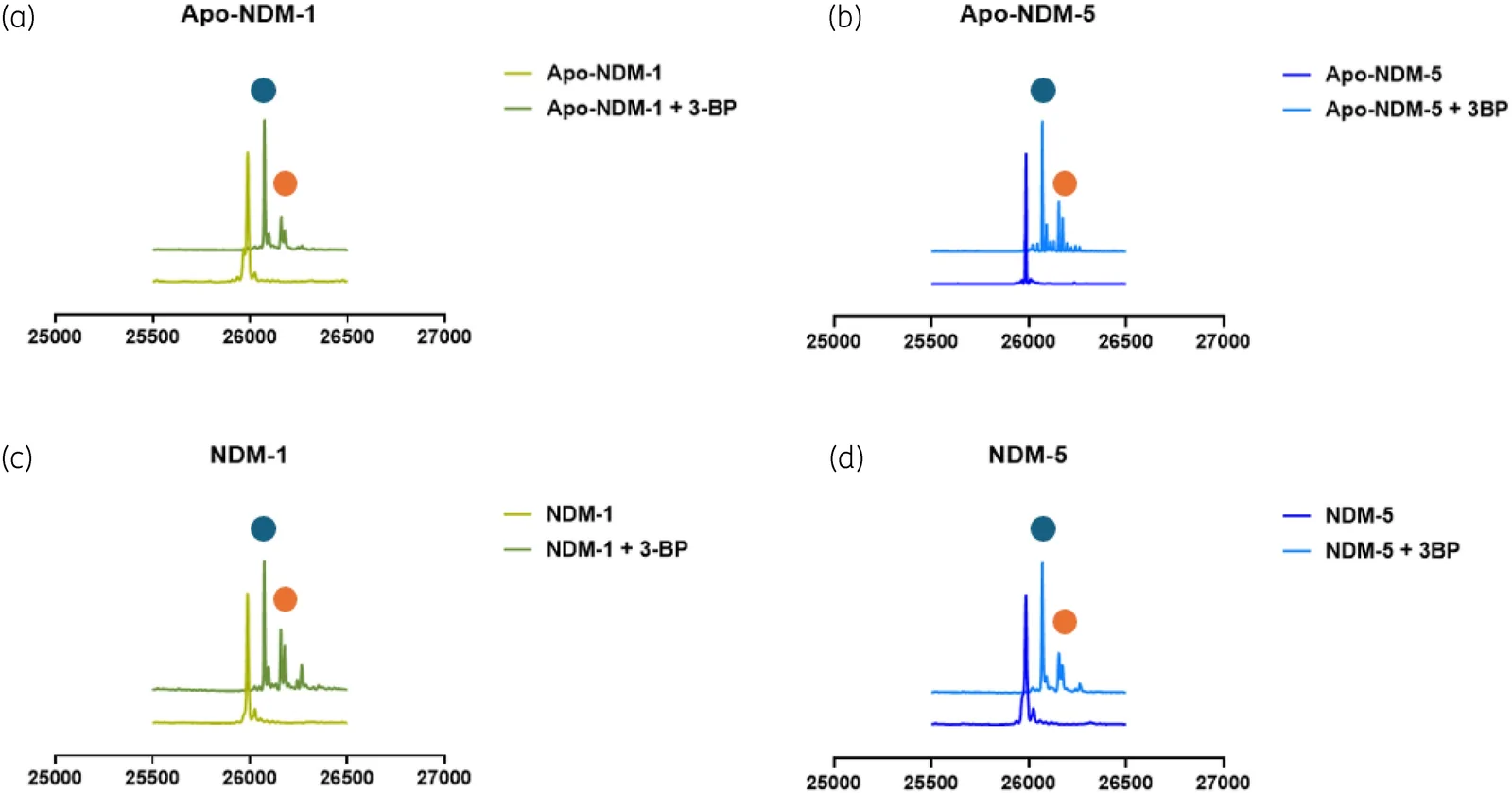
Denaturing MS results of apo- NDM-1/NDM-5 (a, b) and in the presence of Zn(II) (c, d). Control spectra without 3-BP present is shown as the baseline. NDM-1 and NDM-5 have observed masses of 25 987and 25 986 Da. A thioether adduct **6** was formed with NDM-1 and NDM-5 in both the apo- and zinc metalled enzymes highlighted by the blue circle (+87 Da). The presence of additional peaks (orange circle) is proposed to be an intact tetrahedral hemithioketal with fragments identified as +166 and +168 Da, proposed to be ^79^Br and ^81^Br isotopes in the intact 3-BP structure **4**. An additional fragment at +171 Da was also observed, potentially also being the intact adduct or the formation of two alkylation events. A fragment at +281 Da was unable to be identified in NDM-1 and was assigned as an artefact.

Ambler class B1 MBLs contain two Zn (II) cofactors bound in the zinc-1 and zinc-2 sites where the latter is reported to show reduced Zn (II) binding affinity compared to that found in the with zinc-1 site.^40–42^ This zinc-2 site contains a conserved cysteine residue (Cys_208_ in NDM-1), therefore, if the zinc-2 site becomes vacant upon dissociation of the Zn(II) metal ion, a free thiol on the cysteine would become exposed and be accessible to modification by 3-BP.^43^ 3-BP was tested against both the natural- and apo- (non-zinc containing) form of purified MBLs, NDM-1 and NDM-5, each of which contain only one cysteine, under denaturing MS conditions. 3-BP reacted with both NDM-1 and NDM-5 regardless of whether Zn(II) was present (Figure 3).

**Figure 4.**
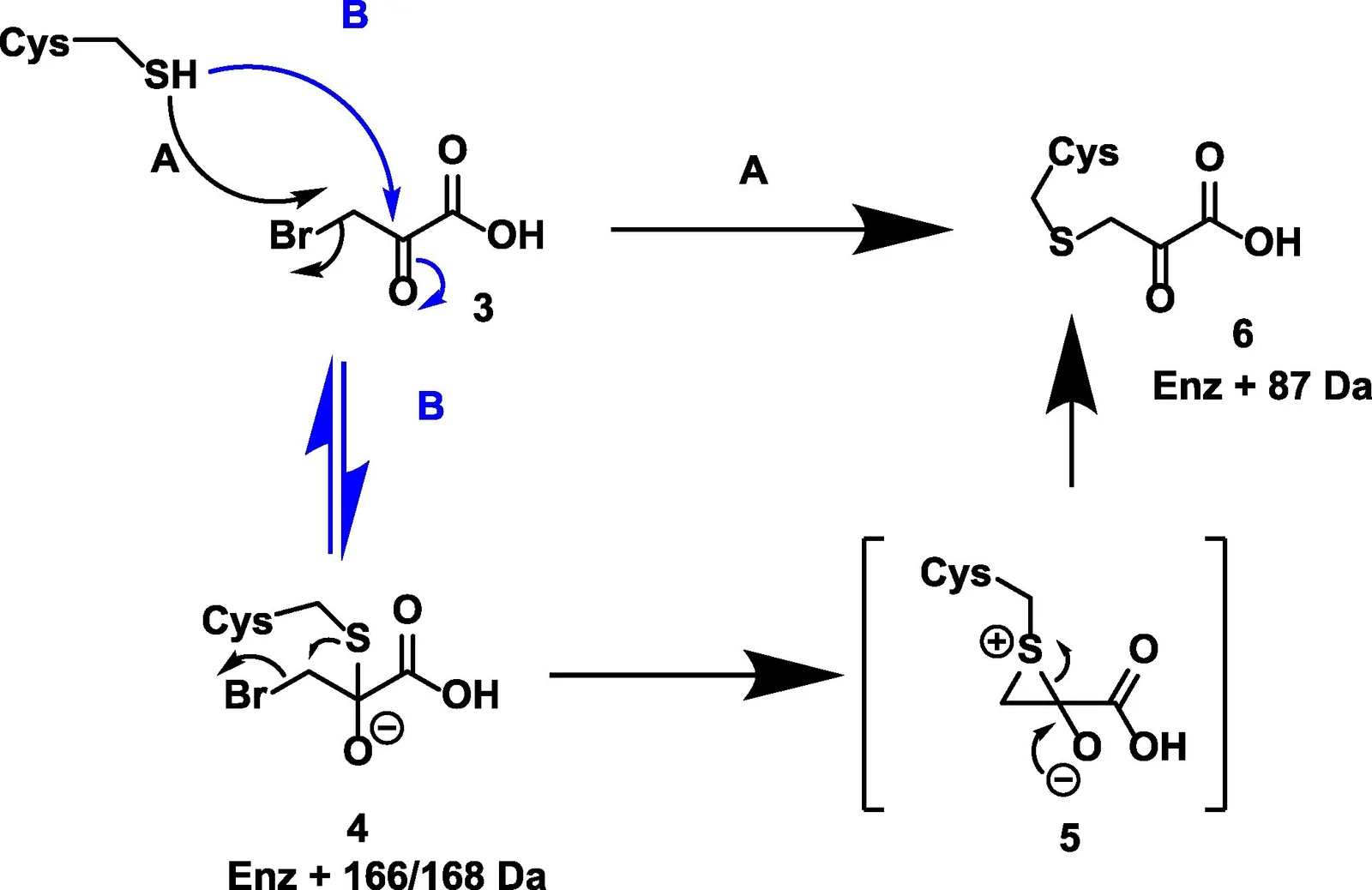
Possible pathways for the reaction of 3-BP covalent bond formation with active site cysteine residue. Pathway **A** shows S_N_2 displacement of the bromide giving a thioether. Pathway **B** shows initial reaction at the ketone, followed by rearrangement to give the thioether adduct. Possible MS fragments noted their enzyme + conjugate fragment.

The observed mass for NDM-1 and NDM-5 was found to be 25 987 and 25 986 Da respectively and in good agreement with the calculated mass of 25 988 and 25 984 Da. It was noted that as denaturing MS conditions were used, only apo-proteins were observed. On the addition of 3-BP to both the apo- and metallated NDM-1 and NDM-5, the major fragment observed was a +87 Da fragment, resulting in a mass (NDM-1 = 26 074 Da, NDM-5 = 25 986 Da) corresponding to a covalent reaction with 3-BP, resulting in HBr loss and the formation of covalent adduct 6 (Table S3). In both enzymes, additional fragments were also observed of +166 and +168 Da, potentially corresponding to the ^79^Br and ^81^Br isotopes of an intact covalent 3-BP adduct (26 153 and 26 155 Da in NDM-1, 26152 and 26 154 Da in NDM-5). Although further work is required the additional fragments may correspond to a hemithioketal (4), consistent with an initial reaction with the ketone of 3-BP (Figure 4).^44^

Similar observations have been made with the reactions of halomethyl ketones against other nucleophilic cysteine containing proteins, with crystallography data supporting the formation of a hemithioketal intermediate.^44–47^ Previously, it was proposed that 3-BP forms a reversible covalent adduct with NDM-1 via pathway **A**; it is, however, generally considered that reactions of halomethyl ketones (such as 3-BP) with cysteines are irreversible, due to the formation of a stable thioether bond.^20,44,47^ As a result, it is proposed that pathway **B** and the formation of the intermediate hemithioketal is more likely to reflect an initial reversible covalent reaction previously reported, followed by irreversible rearrangement to give a thioether.^48,49^

3-BP displayed here shows solid foundation to be built on, showing evidence that MBLs can be targeted covalently and may allow for the development of future clinically useful MBL inhibitors. In summary, 3-BP was shown to inhibit NDM-1 in *A. baumannii* and *K. pneumoniae* and NDM-5 in *E. coli* isolates, expanding on previous findings.^20^ Microbiological studies showed that 3-BP alone did not have any intrinsic activity but restored the activity of meropenem (2 mg/L) against a total of eight resistant MBL producing clinical or environmental isolates of *E. coli*, *K. pneumoniae* and *A. baumannii* when co-administered. 3-BP failed to restore the activity of amoxicillin (8 mg/L) against five SBL producing isolates of *E. coli*, *K. pneumoniae* and *E. cloacae*, reinforcing the potential of 3-BP as a selective MBL inhibitor. The parent scaffold, pyruvic acid showed no inhibitory activity against MBL or SBL producing isolates, meaning the inhibitory activity of 3-BP against MBLs came from the addition of bromine to the scaffold and the introduction of covalent interactions with the MBL active site cysteine.

MS studies evidence a covalent reaction between 3-BP and an active site cysteine to irreversibly give a thioether, potentially via a reversibly formed hemithioketal. 3-BP is a hexokinase inhibitor and despite its reactivity has been explored as a potential anti-cancer treatment.^25–27^ Studies with mouse fibroblastic cells indicate 3-BP has low cytotoxicity at up to 200 μM.^20^ Therefore, although its reactivity may preclude its development, it may inspire the development of covalently reacting inhibitors for MBLs, an inhibition strategy that has been successful for SBL inhibitors. Probably reflecting the sometimes-advantageous properties of covalent inhibition, including prolonged target inactivation and, in many cases, irreversible, enabling efficient substrate competition.^50–54^

## Supplementary Material

dlag177_Supplementary_Data

## Supplementary data

Figure S1 and Tables S1 to S3 are available as Supplementary data at *JAC-AMR* Online.
